# Sexual risk behaviors, mental health outcomes and attitudes supportive of wife-beating associated with childhood transactional sex among adolescent girls and young women: Findings from the Uganda Violence Against Children Survey

**DOI:** 10.1371/journal.pone.0249064

**Published:** 2021-03-25

**Authors:** Laura Chiang, Ashleigh Howard, Kirsten Stoebenau, Greta M. Massetti, Rose Apondi, Jennifer Hegle, Mondo Kyatekka, Caroline Stamatakis, Lydia Wasula, George Aluzimbi

**Affiliations:** 1 National Center for Injury Prevention and Control, Centers for Disease Control and Prevention, Atlanta, GA, United States of America; 2 Strategic Innovative Solutions, LLC, Clearwater, FL, United States of America; 3 School of Public Health, University of Maryland, College Park, MD, United States of America; 4 Center for Global Health, Centers for Disease Control and Prevention, Atlanta, GA, United States of America; 5 Uganda Ministry of Gender, Labour and Social Development, Kampala, Uganda; Washington University in St. Louis, UNITED STATES

## Abstract

Compared to young men, Ugandan young women are disproportionately impacted by HIV. Childhood transactional sex may contribute to this disparity. Using data from the 2015 Uganda Violence Against Children Survey, we used logistic regression models to assess the association between childhood transactional sex and negative outcomes. Among 18-24-year-old young women who had sex prior to 18 (n = 982), those who ever engaged in transactional sex had 5.9 times [adjusted odds ratio (AOR); confidence interval (CI): 1.6–22.2] higher odds of having multiple sexual partners in the past year; 5.2 times (AOR; CI: 2.1–12.9) higher odds of infrequent condom use in the past year; 3.0 times (AOR; CI: 1.2–7.9) higher odds of hurting themselves intentionally; and 3.2 times (AOR; CI: 1.3–7.7) higher odds of having attitudes justifying spousal abuse than young women who never engaged in transactional sex. Interventions for transactional sex and HIV in Uganda should consider prioritizing prevention, harm-reduction and continued investment in adolescent girls’ and young women’s futures.

## Introduction

Despite progress towards epidemic control, HIV remains a leading cause of death in sub-Saharan Africa (SSA) [1]. Adolescent girls and young women (AGYW) ages 15–24 are disproportionately impacted, accounting for two thirds of new infections in Sub-Saharan Africa and 20% of new infections in Uganda [2–4]. In Uganda, HIV prevalence is almost four times higher among females ages 15–19 and 20–24 than same-aged males [5]. Structural and contextual factors contribute to this marked vulnerability of AGYW, including early marriage, access to alcohol, dropping out of school, economic vulnerability, age disparate sex, early sexual debut, and sexual risk behaviors including transactional sex [6–8].

A growing body of evidence has demonstrated that transactional sex increases AGYW’s risk of HIV in SSA [7, 9–11]. Transactional sex refers to “noncommercial, nonmarital sexual relationships motivated by the implicit assumption that sex will be exchanged for material support or other benefits” [12]. This definition reflects research that differentiates transactional sex from Western connotations of sex work, emphasizing that these transactional sex *relationships* are structured by economic uncertainty and gender inequality within the context of widely-held gender expectations in heterosexual relationships that men should provide for their partners, and that in exchange, women should provide sexual and domestic services [12–14]. Women and girls may engage in these relationships for reasons ranging from securing basic needs to meeting aspirations of improved social status [12]. Studies indicate that women’s agency (capacity to make and act upon decisions [15]) in transactional sex relationships also varies substantially, ranging from cases where women perceive hold little to no power to those where they perceive they hold significant levels of decision-making power and control [12]. Several potential mechanisms increase women’s risk for HIV from transactional sex, including violence [16], having multiple partners and not using condoms [17, 18], alcohol misuse [19], and age-disparate transactional relationships [20–23].

Research from Uganda points to similar patterns, emphasizing the central importance of male provision in all relationships, and expected access to women’s sexuality in return [19, 24]. AGYW’s reported level of agency within transactional sex relationships in Uganda also varies. In some studies, they describe themselves as savvy and powerful, able to extract money without having sex, or to benefit from having multiple partners [25–27]. In other research, AGYW are described as having very little power in transactional sex relationships, being forced or coerced into sex, often by older, wealthy men [28] in positions of power, such as teachers [29, 30], as well as by peers [24].

Researchers in the field largely agree that engagement in transactional sex as a child constitutes sexual exploitation [31]. The potential exploitative and coercive aspects of transactional sex in childhood are important to consider. Age disparities, in addition to underlying gender inequality within relationships, reduce girls’ negotiating power within the context of sex [21, 24]. Money and other forms of support are difficult for many girls to decline, particularly in resource-poor settings and when offered by older men in positions of power [21, 30, 32]. Some girls who receive support from men may do so with full awareness for the terms of engagement [26, 29, 33] in the Ugandan context. Although there has been considerable improvement in programming aimed at reaching AGYW with sexual risk reduction messages, abstinence-only education remains the norm in Ugandan schools [34], and access to sexual and reproductive health information and services are restricted for unmarried youth [35]. Consequently, some young girls who receive gifts from men may not fully understand the expectations or potential consequences, others may only later be pressed into sex by men who began supporting them without ‘strings attached’ [36].

Research has documented the determinants for engaging in transactional sex and its association with HIV, but very little research has addressed the consequences of early transactional sex on later risk behaviors, mental health, and attitudes supportive of wife-beating. The purpose of the present study is to assess the associations of transactional sex in childhood among AGYW in Uganda with negative outcomes including mental and physical health consequences, attitudes supportive of wife-beating, and sexual risk behaviors in young adulthood, with a particular lens towards understanding how transactional sex in childhood may increase risk of HIV later in life.

## Methods

The 2015 Uganda Violence Against Children Survey (VACS) was a national household survey led by the Uganda Ministry of Gender, Labour and Social Development with financial support from the President’s Emergency Plan for AIDS Relief (PEPFAR) and technical and implementation support from the Uganda Bureau of Statistics, the AfriChild Centre for Excellence, ChildFund, Makerere University, TPO Uganda, UNICEF, United States Agency for International Development (USAID) and United States Centers for Disease Control and Prevention (CDC). For the survey, 13-24-year-olds were interviewed about lifetime and current experiences of violence and topics including HIV testing history and status, sexual risk behaviors and transactional sex. A multi-stage, geographically clustered sample design yielded nationally representative estimates; AGYW were oversampled in geographic areas with high HIV burden to understand geographic patterns of violence and HIV programming in those areas [37, 38]. Households and participants were randomly selected for participation and there were no incentives offered. A split-sample design included enumeration areas sampled separately for females and males. A total of 368 enumeration areas in the female sample yielded 3,159 completed questionnaires with an overall response rate of 82.2%. Data collection occurred September-November 2015. Interviewers were recruited that had prior experience with survey research but were generally younger (20–35 years old) to facilitate rapport with the participants. They were trained on all study protocols, including ethical protections, and participated in field practice prior to the start of data collection. Responses were recorded electronically, using netbooks with data collection tools programmed in CSPro. The survey was administered in eight commonly-spoken languages used in national surveys as recommended by the Uganda Bureau of Statistics: *Ateso*-Karamajong, English, Luganda, Lugbara, Luo, Runyankole-Rukiga, Runyoro-Rutoro, and Swahili and interviewers were recruited who spoke one or more study languages.

The VACS questionnaire was designed by a group of scientists and public health professionals with subject matter expertise in violence, drawing on the literature and validated violence measures. The questionnaire was further adapted by Ugandan stakeholders to the local context. The Uganda VACS questionnaire can be found on the Together for Girls website (togetherforgirls.org). The study was reviewed and approved by Institutional Review Boards at the Makerere University School of Public Health in Uganda and the CDC. The study followed best practices for violence surveys [39, 40]. Every participant provided informed assent (for minors) or consent (for young adults), including parental consent for those under 18 years of age. The Uganda VACS used an oral consent process for two primary reasons. The primary reason for an oral consent is because the sensitive nature of the study and the need to avoid leaving any hardcopy information behind that could put a participant at risk from a perpetrator in the household or community. Second, due to possible illiteracy in the survey population, it was determined that an oral consent was the most appropriate for the study setting and age. Oral consent was documented by interviewers in the electronic data collection form. The Makerere University School of Public Health and the CDC IRBs both approved oral consent. Interviews were conducted in private to ensure confidentiality and facilitate disclosure.

Those participants who met study-specified criteria were offered a voluntary direct referral to a qualified counselor who could also facilitate linkage to additional services. These criteria included any participant who (1) had experienced recent violence, (2) exchanged sex for money or goods in childhood, (3) became emotionally upset during the interview, (4) reported feeling unsafe in their current living situation, (5) reported being in immediate danger, or (6) asked for violence services. All such referrals were coordinated by the Uganda Transcultural Psychosocial Organisation. Additional details regarding the methodology and procedures (including sample size and power calculations) for the VACS Uganda are available in the Uganda VACS report [41].

In an attempt to separate out the exposure to childhood transactional sex and the outcomes of interest, analyses were restricted to 18-24-year-olds, and most outcome variables were asked about the past 12 months or the past 30 days, allowing for a temporal separation between exposure (childhood transactional sex) and outcomes.

### Measures

#### Childhood transactional sex

Participants were asked if they ever had sex with each of their three most recent sex partners or someone else “because this person provided you with material support or help in any other way? Material support means helping you pay for things or giving you gifts or other things such as food, school fees or money.” Participants who said ‘yes’ were asked questions about their age the first time this happened. Participants were also asked whether the partner with whom they had transactional sex was younger, about the same age, less than 5 years older, 5–10 years older, or more than 10 years older than they were at the time of first sex with that partner. Analyses were restricted to transactional sex that occurred prior to age 18 (childhood transactional sex).

#### Socio-demographics

Dichotomous variables were created for marriage or cohabitation status (ever married or cohabitated vs. never), pregnancy history (ever pregnant vs. never), and orphan status (single or double orphan prior to age 18 vs. not an orphan). Other socio-demographic variables included religion (Catholic, Protestant, Muslim, or other, including Seventh-Day Adventist and Pentecostal), participant age (divided into 3 age bands: 18–20, 21–22, 23–24), and educational attainment (less than primary school; completed primary school; completed secondary school; higher than secondary school). Seven items were used to calculate the Simple Poverty Scorecard™ Uganda [42], which estimates the likelihood of poverty in a household, following methods by Miller, Chiang and Hollis [43]. Points were allocated to each of the indicator questions, summed for an overall score, and assigned to economic status tertiles.

Health risk behaviors and outcomes of childhood transactional sex included sexual risk behaviors, mental health problems, substance misuse, attitudes about violence and gender, and intimate partner violence victimization in young adulthood and lifetime perpetration and the measures are described below.

#### Sexual risk behaviors

Dichotomous variables reflected no or infrequent self-reported condom use in the past 12 months, multiple (having 2 or more) sex partners in the past 12 months, never tested for HIV, and self-reported HIV positive status at the most recent HIV test. Self-reported HIV status is an imperfect measure for a number of reasons, but we included it in the analysis since HIV biomarker data was not collected.

#### Mental health problems

Dichotomous variables reflected current severe or moderate/severe mental distress using the Kessler 6 scale [44] that measures anxiety and depression. The Kessler 6 is a brief measure that has been show to effectively and efficiently differentiate DSM-IV from non DSM-IV cases and has been widely used in a number of household surveys in a number of settings, including the WHO the WHO World Mental Health Surveys [45]. Individuals who scored 10 or higher on the scale were considered to be demonstrating severe mental distress and those with a score of four or higher were considered to have moderate/severe distress., Additionally, the survey measured lifetime suicidal ideation, attempted suicide among those with ideation, and lifetime self-harm.

#### Substance misuse

This composite variable measured any excessive alcohol use (having been drunk) in the past 30 days, any drug use in the past 30 days, and any tobacco use in the past 30 days. The composite variable was created because there were too few responses to assess drug use or tobacco use independently. The drug use question asked about “drugs such as marijuana, pills, ecstasy/E, cocaine, “brown sugar”/heroin, Kubba, Khat, or sniffed any chemical such as petrol or glue” in the past 30 days.

#### Attitudes supportive of wife-beating

This variable was adapted from a Demographic and Health Survey question about attitudes regarding domestic violence in five scenarios. The five scenarios included for VACS were that a man is justified in beating his wife if she (1) goes out without telling him, (2) does not take care of the children, (3) argues with him, (4) refuses to have sex with him, or (5) burns the food. Endorsement of one or more scenarios is considered to be an attitude supportive of wife-beating.

#### Intimate partner violence victimization in young adulthood

This included intimate partner sexual violence or intimate partner physical violence victimization after age 18.

#### Lifetime violence perpetration

This included two different measures of perpetration. (1) ever sexual or physical violence perpetration towards an intimate partner; (2) or ever sexual or physical violence perpetration towards a non-intimate partner.

### Statistical analysis

The prevalence of childhood transactional sex, age of first transactional sex experience, and age differences between female and male transactional sex partners were estimated along with 95% confidence intervals (CIs). SAS (version 9.4; SAS Institute, Inc, Cary, NC) was used for data management and analysis. All analyses account for the complex survey design and applied sample weights to achieve nationally representative estimates. Sexual risk, attitudes supportive of wife-beating and mental health outcomes of childhood transactional sex were explored by conducting unadjusted and adjusted logistic regression models using the SURVEYLOGISTIC procedure in SAS. Multivariable logistic regressions controlled for ever-married or cohabitated, ever pregnant, household economic status, educational attainment, religion, and orphan status. We also controlled for geographic region (central, eastern, northern, western) when modeling outcomes that were associated with region in bivariable analyses (p<0.05). Odds ratios with a p-value of less than or equal to 0.05 are considered significant and flagged with a double asterisk in the tables. Odds ratios with a p-value greater than 0.05 and less than 0.1 are indicated in the tables with a single asterisk.

Questions included in the present analysis reflect very low levels of missing data (<1% for most questions). Most variables included in the present analysis are comprised of multiple questions and the analytic approach used pairwise deletion to handle missing data. The total denominator is included in the table with the adjusted model to reflect data missingness.

## Results

Out of a total of 1,795 young women ages 18–24, the final analytic sample included 982 who ever had sex prior to age 18. Table 1 includes sociodemographic characteristics of the sample. The majority were married or cohabitating (86.0%; 95% CI: 79.8–92.2) and had been pregnant at least once (82.2%; 95% CI: 75.9–88.4). More than one in ten young women had less than a primary school education (13.6%; 95% CI: 9.2–18.0), 52.6% completed primary school only (95% CI: 44.6–60.6), 31.0% completed secondary school (95% CI: 21.9–40.0) and only 2.8% had any higher education (95% CI: 0.9–4.8). Nearly one third had lost one or both parents in childhood (32.0%; 95% CI: 24.9–39.1). Most females identified as Catholic (37.0%; 95% CI: 29.3–44.7) or Protestant (30.3%; 95% CI: 24.6–36.0), 17.1% identified as Muslim (95% CI: 10.3–23.9), and 15.6% as another religion (95% CI: 8.1–23.1).

**Table 1:** Background characteristics of Ugandan females ages 18–24 who ever had sex prior to age 18, Uganda Violence Against Children Survey 2015.

Total N = 982	Unweighted n	Weighted % (95% Confidence Interval)
*Age*		
18–20	311	33.9 (27.9–39.9)
21–22	419	39.2 (34.2–44.2)
23–24	252	26.9 (20.9–32.8)
	Weighted Mean Age (95% Confidence Interval)	Standard Error of the Mean
*Mean age*	20.7 (20.4–21.0)	0.1
	Unweighted n	Weighted % (95% Confidence Interval)
Ever Married or Cohabitated	889	86.0 (79.8–92.2)
Married before age 18	587	54.4 (48.4–60.4)
Ever Pregnant	863	82.2 (75.9–88.4)
*Economic Status*		
Low	345	35.8 (28.0–43.7)
Middle	346	30.9 (24.1–37.7)
High	291	33.3 (26.0–40.5)
*Educational Achievement*		
Less than Primary School	190	13.6 (9.2–18.0)
Completed Primary School	529	52.6 (44.6–60.6)
Completed Secondary School	241	31.0 (21.9–40.0)
Higher than Secondary School	22	2.8 (0.9–4.8)
Orphan (single or double)	322	32.0 (24.9–39.1)
*Religion*		
Catholic	487	37.0 (29.3–44.7)
Protestant	268	30.3 (24.6–36.0)
Muslim	99	17.1 (10.3–23.9)
Other^2^	127	15.6 (8.1–23.1)
*HIV status*		
Living with HIV^3^	33	3.1 (1.0–5.1)

^1^Standard error of the mean

^2^Other includes Seventh Day Adventist, Pentecostal, and Other

^3^ HIV status is self-reported among those who were ever tested; no testing was conducted for the Uganda VACS

The overall prevalence of childhood transactional sex was 14.8% (95% CI: 10.6–18.9; Table 2). The age of first experience of childhood transactional sex was ages 16–17 years for 85.7% of girls (95% CI: 75.6–95.8) and age 15 or younger for 14.3% (95% CI: 4.2–24.4). For the majority of first childhood transactional sex experiences, the partners were older: 20.4% of partners were more than 10 years older (95% CI: 5.7–35.1), 36.0% were 5–10 years older (95% CI: 16.9–55.0), and 24.3% were less than 5 years older (95% CI: 12.4–36.2). Transactional sex partners were the same age or younger for fewer than 20% of girls (19.4%: 95% CI: 6.9–31.9).

**Table 2:** Prevalence and characteristics of first experiences of childhood transactional sex among females ages 18–24 who ever had sex prior to age 18, Uganda Violence Against Children Survey 2015.

	Weighted % (95% Confidence Interval)
Childhood transactional sex (n = 122)	14.8% (10.6–18.9)
Age at first Transactional Sex	
15 or younger (n = 33)	14.3% (4.2–24.4)
16–17 (n = 89)	85.7% (75.6–95.8)
Age of First Transactional Sex Partner	
Same age or younger than respondent (n = 22)	19.4% (6.9–31.9)
Less than 5 years older (n = 42)	24.3% (12.4–36.2)
5–10 years older (n = 41)	36.0% (16.9–55.0)
More than 10 years older (n = 15)	20.4% (5.7–35.1)

Associations between childhood transactional sex and sexual risk and other outcomes are presented in Table 3. Unadjusted analyses indicated an association between childhood transactional sex and no or infrequent condom use in the past 12 months, having multiple sex partners in the past 12 months, any sexual or physical violence perpetration towards a non-partner, and endorsement of attitudes supportive of wife-beating. Childhood transactional sex was not associated with never testing for HIV, suicidal ideation, sexual intimate partner victimization after age 18, physical intimate partner violence victimization after age 18, any sexual or physical violence perpetration towards a partner, self-reported HIV status, current moderate or severe mental distress, attempted suicide, and substance misuse in the past 30 days in the unadjusted analyses.

**Table 3:** Health behaviors and health outcomes among 18–24 year-old females in Uganda who ever had sex before age 18, by history of childhood transactional sex.

	No childhood transactional sex N = 857	Experienced childhood transactional sex N = 122		
	Weighted % (95% CI)	Weighted % (95% CI)	Crude Odds Ratio (95% CI)	Adjusted Odds Ratio (95% CI)
**Sexual risk behaviors by experience of childhood transactional sex**				
No or infrequent condom use in the past 12 months^1^ (N = 827)^2^	36.3 (29.3–43.3)	71.3 (55.4–87.1)	4.3 (1.8–10.5) p = 0.001	**5.2 (2.1–12.9)**^**3**^ **p = 0.0004**
Multiple sex partners in the past 12 Months^1^ (N = 829)^2^	6.4 (3.1–9.6)	23.9 (1.8–45.9)	4.6 (1.2–17.4) p = 0.03	**5.9 (1.6–22.2)**^**3**^ **p = 0.008**
HIV positive^4^ (N = 860)^2^	2.8 (0.7–4.8)	5.3 (0.0–13.4)	2.0 (0.3–11.8) p = 0.4	1.4 (0.3–6.2)^5^ p = 0.6
Never tested for HIV (N = 933)^2^	8.7 (4.1–13.3)	19.0 (0.0–39.6)	2.4 (0.9–6.8) p = 0.08	1.6 (0.5–4.7)^**3**^ p = 0.4
**Mental health outcomes by experience of childhood transactional sex**				
Moderate/severe mental distress in past 30 days (N = 933)^2^	51.4 (43.3–59.4)	50.1 (32.6–67.5)	0.9 (0.4–2.2) p = 0.9	0.9 (0.4–2.1)^5^ p = 0.8
Severe mental distress in past 30 days (N = 933)^2^	13.6 (9.2–18.1)	18.0 (3.8–32.2)	1.4 (0.5–4.0) p = 0.5	0.8 (0.2–3.2)^5^ p = 0.7
Suicidal ideation (N = 932)^2^	18.6 (13.1–24.1)	31.9 (15.4–48.3)	2.0 (1.0–4.3) p = 0.06	1.4 (0.6–3.2)^**3**^ p = 0.5
Attempted suicide among those who thought of suicide (N = 163)^2^	38.4 (22.0–54.7)	43.6 (14.5–72.7)	1.2 (0.3–4.9) p = 0.8	4.3 (0.8–22.1)^5^ p = 0.8
Intentional self-harm (N = 933)^2^	8.7 (4.7–12.7)	18.1 (5.8–30.4)	2.3 (0.9–6.0) p = 0.09	**3.0 (1.2–7.9)**^5^ **p = 0.02**
**Substance misuse use by experience of childhood transactional sex**				
Substance misuse past 30 days (drugs, tobacco, alcohol) (N = 933)^2^	15.8 (10.4–21.1)	14.3 (5.6–23.9)	0.9 (0.4–2.0) p = 0.8	0.8 (0.3–2.0)^5^ p = 0.6
**Violence victimization after age 18 by experience of childhood transactional sex**				
Adult Sexual Intimate Partner Violence (N = 933)^2^	22.3 (16.9–27.8)	41.9 (21.8–62.1)	2.5 (1.0–6.5) p = 0.06	2.4 (0.8–6.7)^**3**^ p = 0.07
Adult Physical Intimate Partner Violence (N = 930)^2^	29.5 (23.1–35.8)	22.4 (10.6–34.1)	0.7 (0.4–1.3) p = 0.3	0.8 (0.3–1.9)^5^ p = 0.6
**Violence perpetration at any age by experience of childhood transactional sex**				
Perpetrated physical or sexual violence towards an intimate partner (N = 930)^2^	10.9 (6.9–14.9)	16.5 (5.5–27.5)	1.6 (0.7–3.8) p = 0.3	1.6 (0.6–4.5)^5^ p = 0.3
Perpetrated physical or sexual violence towards a non-partner (N = 933)^2^	13.9 (8.6–19.2)	31.3 (16.2–46.4)	2.8 (1.2–6.5) p = 0.01	2.0 (0.8–4.7) ^**3**^ p = 0.1
**Current attitudes supportive of wife-beating by experience of childhood transactional sex**				
Attitudes Supportive of Wife-beating (N = 933)^2^	60.1 (52.2–68.0)	78.7 (65.2–92.2)	2.4 (1.2–5.1) p = 0.02	**3.2 (1.3–7.7)**^**3**^ **p = 0.009**

Odds ratios with p≤ = 0.05 are highlighted in bold font for adjusted odds ratios

1. Calculated among those who had sex in the past 12 months

2. N is the denominator in the final adjusted model

3. Model includes economic status, education, religion, orphan status, ever married or cohabitated, ever pregnant, and region

4. HIV status is self-reported among those who were ever tested; no testing was conducted for the Uganda VACS

5. Model includes economic status, education, religion, orphan status, ever married or cohabitated, and ever pregnant

After adjusting for all covariates, childhood transactional sex was significantly associated with 5.9 times [adjusted odds ratio (AOR); 95% CI: 1.6–22.2] higher odds of having multiple sexual partners in the past year. Similarly, those who engaged in childhood transactional sex had 5.2 times (AOR; 95% CI: 2.1–12.9) higher odds of never or infrequently using condoms in the past year. We also found that childhood transactional sex was significantly associated with 3.0 times (AOR; 95% CI: 1.2–7.9) increased odds of intentional self-harm. Finally, compared to 18-24-year-olds who had never engaged in childhood transactional sex, those who had engaged in childhood transactional sex had 3.2 times (AOR; 95% CI: 1.3–7.7) higher odds of attitudes supportive of wife-beating. Childhood transactional sex was not significantly associated with sexual or physical violence perpetration towards a non-partner in the multivariate analyses.

## Discussion

This analysis examined the associations between childhood transactional sex and sex risk behaviors, attitudes supportive of wife-beating, substance misuse, violence, and mental health outcomes in Ugandan females ages 18–24. While previous studies have focused on identifying determinants of transactional sex and associations with HIV, this study highlights potential mechanisms explaining the association between early transactional sex and risk of HIV infection and other negative health outcomes in early adulthood. In summary, we found that one in seven 18–24 year old Ugandan young women who had ever had sex before age 18 had also engaged in transactional sex in childhood. The majority of these young women engaged in transactional sex for the first time between the ages of 16 and 17 and their first transactional sex partners were most often at least five years older. Bivariate analyses found associations between childhood transactional sex and having multiple sex partners in the past 12 months, no or infrequent condom use in the past 12 months, violence perpetration towards a non-partner, and attitudes supportive of wife-beating. After adjusting for demographic factors, childhood transactional sex was significantly associated with having multiple sex partners, no or infrequent condom use, attitudes supportive of wife-beating, and intentional self-harm.

That reported early engagement in transactional sex is associated with more recent sexual risk behaviors (i.e. no or infrequent condom use and multiple sex partners in the past 12 months) may be in part explained by gender norms and beliefs that underlie transactional sex [46]. Transactional sex stems from broader gender expectations of relationships within patriarchal societies–that men are expected to provide for their partners and women, in turn, to provide sexual services [47]. A recent pilot study conducted in central Uganda examined whether there are gender norms attached to this broader expectation that may explain young women’s risk of HIV through transactional sex. The study found evidence for a higher social acceptance of men’s control over sexual decision-making power in relationships when they provide more material support. The study also found evidence for increased peer acceptance of young women taking on an additional sexual partner if their primary partner is perceived as not providing enough material support [27]. While the Uganda VACS did not have measures to allow replication of this study, this pilot study provides insight that women who engage in relationships motivated by male provision may be more likely to adhere to expectations that men should control sexual decision-making, including whether or not to use condoms. Girls who adhere to patriarchal beliefs associated with provision by male partners may also be more likely to adhere to other gender inequitable beliefs, including the acceptance of wife-beating. Early and continued engagement in transactional sex can for some young women lead ultimately to more formal sex work, which holds significant social and health consequences [48] and could explain some of the mental health consequences we identified in unadjusted and adjusted analyses.

While being in relationships where material provision takes place is normative in this context [49, 50], new research has demonstrated childhood transactional sex with an age-disparate partner who is 10 or more years older is considered exploitative of the girl child in Ugandan society [51]. Given that one in five females in this study who had childhood transactional sex had a partner 10 or more years older, strategies that address age-disparate transactional sex could be effective in promoting HIV prevention and overall well-being of young women. For example, the Fataki campaign in Tanzania used radio spots to address inter-generational sex and HIV risk and was effective in reducing men’s engagement in age-disparate transactional sex [52]. Adaptation of such effective programs into new contexts, such as Uganda, can provide models for health promotion and HIV prevention. However, as a study from Tanzania shows, community perceptions of non-age-disparate transactional sex relationships can frame them as exploitative against the male partner where the girl or young women is perceived as ‘using’ her partner [53], pointing to the importance of critically addressing gender norms and beliefs attached to male provision in relationships [54].

Transactional sex in adolescent girls and young children can be prevented by deliberate action to understand and respond to its root causes. Uganda has embraced broad violence prevention interventions, some of which are included in the INSPIRE technical package–a multi-sectoral approach of evidence-based strategies to prevent and respond to violence against children [55]. PEPFAR implements DREAMS (**D**etermined, **R**esilient, **E**mpowered, **A**IDS-free, **M**entored and **S**afe) in Uganda, which is focused on preventing HIV among AGYW. DREAMS uses a three-prong ecological model focused on the individual and her sexual partner(s), her family, and the community. The DREAMS model utilizes a multi-sectoral approach of implementing layered evidence-based interventions to reduce AGYW vulnerability to HIV and to increase AGYW agency. Given the focus on empowerment of AGYW and sexual risk reduction, DREAMS could reduce transactional sex and its associated risks, though this has not been evaluated [6].

Access to goods or money is a key motivator for engaging in transactional sex, and prevention programming must take into account the economic opportunity of sex for young people and offer an alternative [46]. Economic strengthening could be important in both preventing entry into transactional sex and giving AGYW engaging in transactional sex an alternative source of income [56]. Prior research points to an inverse relationship between transactional sex and girls’ receipt of “pocket money” from parents or guardians [27, 57]. More importantly, programming that alleviates poverty through opportunity for educational achievement may result in delayed sexual debut and prevention of transactional sex [58, 59]. Post-secondary education has been associated with decreased risk of transactional sex in some settings [60, 61] and there is strong evidence that keeping girls in school reduces HIV risk and may have additional positive outcomes [62–64]. Programming focused on keeping girls in school or getting those who have dropped out back into school may prevent or reduce transactional sex in Uganda.

Prevention efforts need to be adapted for adolescent girls under 18 and take into consideration their cognitive development, life experience, and socioeconomic status, as well as societal gender norms. Parenting and caregiver support programs implemented in community settings can reduce risk for violence and risky sexual behaviors [65, 66]. These interventions that educate parents and caregivers may have potential for reducing transactional sex by increasing protection in families, strengthening family communication, and reducing risky behaviors. Complementing primary prevention of transactional sex with strategies that help AGYW who have already begun engaging in transactional sex to reduce sexual risk behaviors is important to reduce harm and minimize HIV risk [54]. In addition, comprehensive sexual and reproductive health services including access to condoms, education and support for reducing the number of sexual partners, sexually transmitted infection testing and treatment, Pre-Exposure Prophylaxis (PrEP), and family planning are critical tools for harm reduction among AGYW who engage in transactional sex. It is important to note that the study found a significant proportion of the study population was married. Interventions focused on young women should take this context into account.

As the results from this study demonstrate, childhood transactional sex is associated with self-harm in Uganda. Studies focused on transactional sex in adult populations have similarly documented high levels of trauma [67]. HIV prevention programming with AGYW may also benefit from a trauma-informed approach and mental health services for AGYW who have or are still engaging in transactional sex may be an important service. However, we acknowledge that access to high-quality mental health services in LMICs remains a public health challenge [68].

This study has several limitations. First, the VACS is a retrospective study based on self-reports. As such, both recall bias and social desirability may lead to possible under-reporting of key variables included in the present analysis. Additionally, VACS is a cross-sectional survey and therefore it is not possible to assume causation between transactional sex and the outcomes of interest. It is possible that there are common factors that underly both transactional sex in childhood and the outcomes. However, the analysis does have temporal separation between the exposure variable of transactional sex in childhood and time-bound outcome variables including experiences of adult IPV, mental distress, substance use, beliefs about wife beating, infrequent or low condom use and multiple sex partners in the past 12 months (consider a footnote here that it is possible experiences in the last 12 months for 18-year-olds could overlap with childhood TS). The outcome variables of violence perpetration, suicidal ideation and attempts, self-harm, substance use, HIV testing, and HIV status were measured for the lifetime and thus do not allow temporal separation. Future longitudinal research could allow for a more definite understanding of causality. Finally, the sample size may be a limiting factor in detecting significant relationships between childhood transactional sex and outcomes of interest.

## Conclusion

Despite considerable progress over the past few decades, HIV remains a public health crisis in Uganda and AGYW are at considerable risk. Transactional sex in childhood is not uncommon among girls in Uganda. This in turn exposes AGYW to sexual risk behaviors, endorsement of harmful gender norms, and poor mental health. Policies and programs aimed at preventing Ugandan girls from engaging in transactional sex may contribute to HIV prevention for AGYW. Further, giving AGYW currently engaged in transactional sex sexual and reproductive health education, economic and education opportunities, as well as skills and tools to reduce their risk may empower them to avoid transactional sex or reduce their risk. Engaging families and communities and promoting healthy gender norms may also contribute to reducing risk for transactional sex. Recognizing the significant harm for girls under 18 engaging in transactional sex and their increased risk for HIV, the integration of transactional sex prevention and response interventions into HIV programs is imperative.
